# Long-term outcomes of robot-assisted versus minimally invasive esophagectomy in patients with thoracic esophageal cancer: a propensity score-matched study

**DOI:** 10.1186/s12957-024-03358-w

**Published:** 2024-03-20

**Authors:** Toru Sakurai, Akihiro Hoshino, Kenta Miyoshi, Erika Yamada, Masaya Enomoto, Junichi Mazaki, Hiroshi Kuwabara, Kenichi Iwasaki, Yoshihiro Ota, Shingo Tachibana, Yutaka Hayashi, Tetsuo Ishizaki, Yuichi Nagakawa

**Affiliations:** 1 Department of Gastrointestinal and Pediatric Surgery, Tokyo Medical University, 6-1-1 Nishi-Shinjuku, Shinjuku-ku, Tokyo, 160-0023 Japan; 2 Department of Digestive Surgery, Kohsei Chuo General Hospital, 1-11-7 Mita, Meguro-ku, Tokyo, 153-8581 Japan; 3 Department of Surgery, Toda Chuo General Hospital, 1-19-3 Hon-chou, Toda, Saitama, 335-0023 Japan

**Keywords:** Esophageal neoplasms, Esophagectomy, Robotic surgical procedures, Thoracoscopy, Propensity score

## Abstract

**Background:**

Recently, robot-assisted minimally invasive esophagectomy (RAMIE) has gained popularity worldwide. Some studies have compared the long-term results of RAMIE and minimally invasive esophagectomy (MIE). However, there are no reports on the long-term outcomes of RAMIE in Japan. This study compared the long-term outcomes of RAMIE and MIE.

**Methods:**

This retrospective study included 86 patients with thoracic esophageal cancer who underwent RAMIE or MIE at our hospital from June 2010 to December 2016. Propensity score matching (PSM) was employed, incorporating co-variables such as confounders or risk factors derived from the literature and clinical practice. These variables included age, sex, body mass index, alcohol consumption, smoking history, American Society of Anesthesiologists stage, comorbidities, tumor location, histology, clinical TNM stage, and preoperative therapy. The primary endpoint was 5-year overall survival (OS), and the secondary endpoints were 5-year disease-free survival (DFS) and recurrence rates.

**Results:**

Before PSM, the RAMIE group had a longer operation time (min) than the MIE group (P = 0.019). RAMIE also exhibited significantly lower blood loss volume (mL) (P < 0.001) and fewer three-field lymph node dissections (P = 0.028). Postoperative complications (Clavien–Dindo: CD ≥ 2) were significantly lower in the RAMIE group (P = 0.04), and postoperative hospital stay was significantly shorter than the MIE group (P < 0.001). After PSM, the RAMIE and MIE groups consisted of 26 patients each. Blood loss volume was significantly smaller (P = 0.012), postoperative complications (Clavien–Dindo ≥ 2) were significantly lower (P = 0.021), and postoperative hospital stay was significantly shorter (P < 0.001) in the RAMIE group than those in the MIE group. The median observation period was 63 months. The 5-year OS rates were 73.1% and 80.8% in the RAMIE and MIE groups, respectively (P = 0.360); the 5-year DFS rates were 76.9% and 76.9% in the RAMIE and MIE groups, respectively (P = 0.749). Six of 26 patients (23.1%) in each group experienced recurrence, with a median recurrence period of 41.5 months in the RAMIE group and 22.5 months in the MIE group.

**Conclusions:**

Compared with MIE, RAMIE led to no differences in long-term results, suggesting that RAMIE is a comparable technique.

## Background

The first robot-assisted thoracoscopic transhiatal esophagectomy for esophageal cancer was performed in 2001 by Horgan et al. [1]. Since the use of the robot-assisted transthoracic approach in 2002 by Kernstine et al. [2], several surgeries using this method have been reported [3–6], highlighting its valuable application [7, 8]. Robot-assisted minimally invasive esophagectomy (RAMIE) was compared with minimally invasive esophagectomy (MIE), and the short-term treatment outcomes of RAMIE have been extensively documented [9–11]. Several studies in Western countries and Asia have reported the outcomes of RAMIE [12–16], as well as compared RAMIE with thoracotomy [17] and MIE [18, 19]. However, few long-term outcomes of RAMIE for upper mediastinal lymphadenectomy have been reported. Moreover, to our knowledge, there are no reports on the long-term outcomes of RAMIE in Japan.

Surgery for thoracic esophageal cancer is an invasive procedure involving the digestive system, with a high risk of postoperative complications. Thoracoscopic esophagectomy, developed as a minimally invasive surgery, presents challenges owing to the intricacies of surgical procedures within the confined thoracic cavity, demanding considerable skill. Given the potential benefits of robotic assistance, it can be utilized for this delicate operation in a narrow thoracic cavity; therefore, we performed RAMIE on 53 patients using the da Vinci Si. In this study, the short- and long-term treatment outcomes between RAMIE and conventional MIE were compared to demonstrate the usefulness of RAMIE.

## Methods

### Patients

Robot-assisted thoracoscopic esophagectomy was performed on 53 patients between June 2010 and December 2016. Surgical stability was achieved from the eleventh patient. The initial 10 surgeries were excluded from the analysis owing to non-standardized port positions and surgical procedures. The 43 patients who underwent the procedure following the standardization of the method were assigned to the RAMIE group. The indications of robotic and conventional esophageal cancer surgery (systemic conditions, such as cardiopulmonary function) were as follows: (1) thoracic esophageal cancer (any histological type), (2) age ≤ 80 years, (3) invasive tumor depth ≤ clinical T3 factor (mucosal layer to adventitia) upon preoperative diagnosis (excluding patients who underwent salvage surgery after radical chemoradiotherapy), (4) no medical history of extensive thoracic surgery, and (5) provided written consent to undergo robot-assisted surgery.

**Fig. 1:**
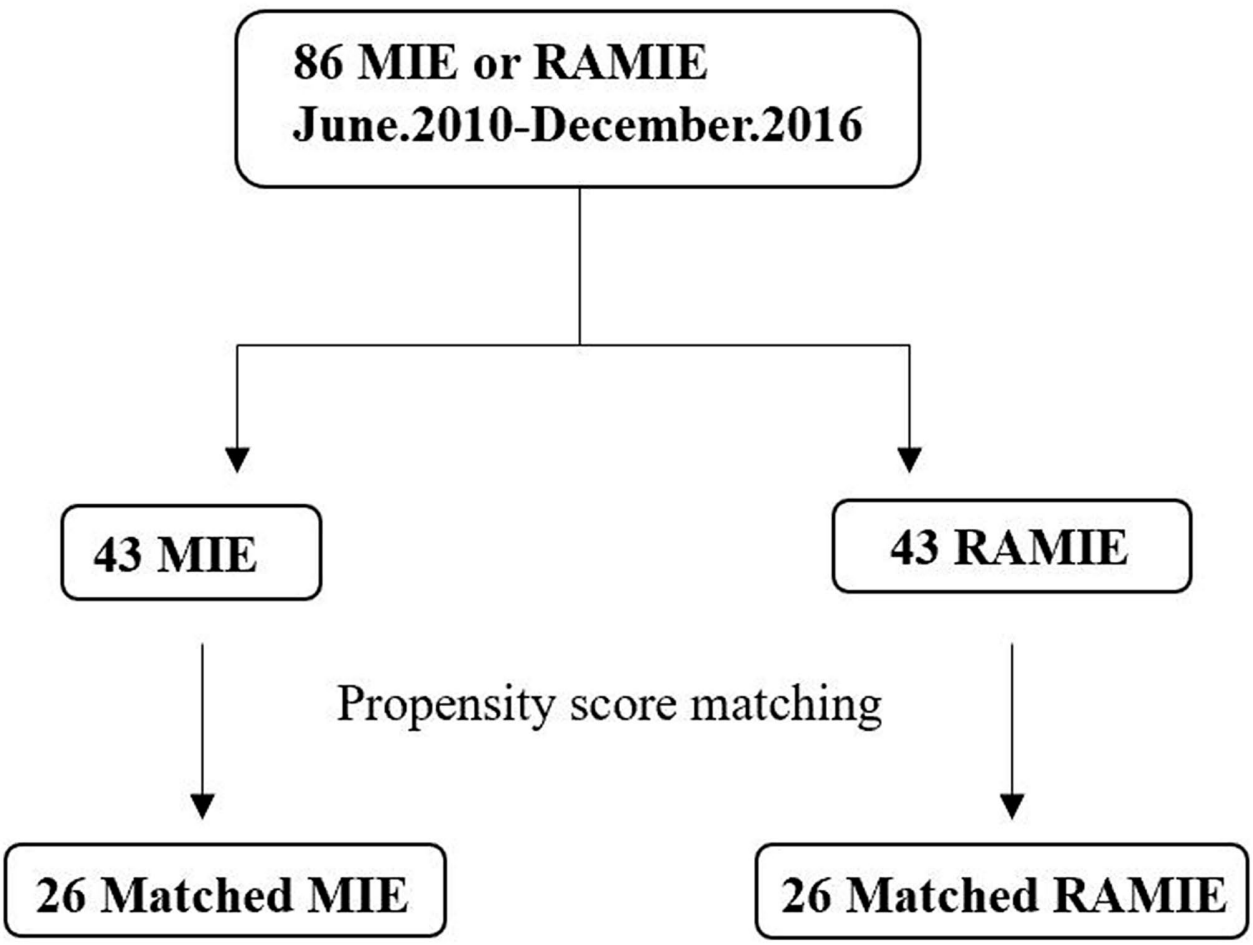
Patient tree of the 86 patients: 43 patients were assigned to each of the MIE and RAMIE groups. MIE: minimally invasive esophagectomy, RAMIE: robot-assisted minimally invasive esophagectomy

In addition, 43 patients who underwent MIE during the same period and met the conditions described above for RAMIE were included in the MIE group (Fig. 1).

The patients selected RAMIE or MIE based on their preferences. At the time of selection, the patients were informed that RAMIE would be performed as part of a clinical study that was approved by the ethics committee of the institution and that MIE was a medical treatment covered by regular health insurance. Surgeries were performed by two surgeons certified by the Japan Robotic Surgery Society. Surgical outcomes, postoperative complications, and long-term treatment outcomes were retrospectively compared between the two groups. This study was approved by the Ethics Committee of Tokyo Medical University (Approval no. 20). Written informed consent was obtained from all the patients who underwent robotic esophagectomy.

### Study endpoints

The short- and long-term postoperative outcomes were compared between the two groups. The primary endpoint was the 5-year overall survival (OS). The secondary endpoints were 5-year disease-free survival (DFS) and recurrence rates.

### Preoperative therapy

Cases receiving preoperative therapy were selected based on JCOG9907 [20]. Neoadjuvant chemoradiotherapy was selected for cases with clinical T3 or higher.

### Surgical procedure (techniques)

Patients underwent RAMIE using the surgical technique previously reported by Osaka et al. and the da Vinci Si Surgical System (Intuitive Surgical, Inc., Sunnyvale, CA) [21]. RAMIE and MIE were performed using a similar surgical procedure. Thoracic surgery was performed under 8-mmHg of artificial pneumothorax in the prone position. The positions of the five ports in RAMIE were as follows: an 8-mm port for the da Vinci right arm slightly ventral to the third intercostal posterior axillary line, a 12-mm port for the da Vinci camera at the sixth intercostal posterior axillary line, an 8-mm port for the da Vinci left arm slightly ventral to the ninth intercostal shoulder blade line, and 12- and 5-mm ports for assistance at the midaxillary line of the fourth and eighth intercostals. The positions of the six ports in the MIE were as follows: a 12-mm port slightly ventral to the sixth intercostal midaxillary line, 12-mm port for the camera at the eighth intercostal shoulder blade line, 12-mm port at the eighth inferior angle of the scapula, and 5-mm ports at the eighth intercostal midaxillary line, sixth medial intercostal scapula, fourth intercostal posterior axillary line, and second intercostal midaxillary line, respectively.

In the upper mediastinum, the lymph nodes around the bilateral recurrent laryngeal nerves (RLN) and esophagus were dissected. In the middle and lower mediastinum, ligation and transection of the arch of the azygos vein and right bronchial artery and dissection of the esophagus and middle and lower mediastinum were performed. The thoracic duct was preserved. For abdominal surgeries, da Vinci was not used in either group. Gastric engorgement and abdominal lymph node dissection were performed using hand-assisted laparoscopic surgery (HALS). Thereafter, the stomach was removed from the body through a small median incision of the HALS. A narrow gastric tube with a width of approximately 3 cm was created and elevated into the cervical region via retrosternal or posterior mediastinal routes. During cervical surgery, the bilateral cervical lymph nodes were dissected, and cervical esophagogastric anastomosis was performed using a circular stapling device. Two- (except #104RL) or three-field lymph node dissection (FLND) was performed based on the degree of disease progression and surgical risks. Three FLND was performed in a patient with upper thoracic esophageal cancer and suspected cervical lymph node metastasis.

### Data collection

We recorded the operation time at each step of the procedure in detail. Thoracic operative time was defined as time from the start of the chest incision to the completion of closure of the thoracic wounds. Intraoperative events such as bleeding, arrhythmia, and iatrogenic injury were recorded. For pathological results, the resected specimens in this study included proximal, distal, and circumferential margins. R0 resection was defined as > 1 mm from all resection margins. R1 resection was defined as microscopical residual tumor, and R2 resection was defined as macroscopical residual tumor. All retrieved lymph nodes were recorded separately for pathological examination. Posttreatment follow-up was performed in our hospital once every 3 months within the first to five years. Postoperative mortality was defined as death from any cause. Postoperative complications including pulmonary complications, cardiac complications, wound infection, bleeding, anastomotic leakage, chylothorax, and RLN paralysis were graded according to the Clavien-Dindo classification [22]. Furthermore, anastomotic leakage, chylothorax, and RLN paralysis were graded according to definitions stated by the Esophagectomy Complications Consensus Group (ECCG) [23].

### Propensity score matching

Propensity score matching (PSM) between patients who underwent RAMIE and MIE was conducted to minimize the selection bias arising from a retrospective study. Co-variables used for PSM included confounders or risk factors based on the literature and clinical practice: age, sex, body mass index, alcohol consumption, smoking (Brinkman Index), American Society of Anesthesiologists stage, comorbidities (myocardial infarction, heart failure, cerebrovascular disease, chronic pulmonary disease, liver disease, diabetes without chronic complications, renal disease), tumor location, histology, clinical TNM stage (Union for International Cancer Control, 7th edition), and preoperative therapy. Propensity scores for each patient were obtained using a multivariate logistic regression model based on patient characteristics. Nearest-neighbor matching was performed using a caliper width of 0.2 standard deviations of the estimated propensity score logit for one-to-one pair matching without replacement. The remaining propensity-matched groups were assessed using the standardized mean difference (SMD), with absolute values < 0.1 considered well-balanced between the two groups.

### Statistical analysis

Statistical analyses were performed using SPSS version 28.0 software (IBM Corp., Armonk, NY, USA). Survival analysis was performed using the Kaplan–Meier method. Patients in the two matched cohorts were reviewed for OS and DFS. Continuous variables were presented as SMD and median (interquartile range). Categorical variables were presented as numbers (%).

Outcomes in the matched cohorts were compared using the McNemar test or McNemar’s exact test for categorical variables and the Wilcoxon signed-rank test for continuous variables. In the pre-matched cohorts, these outcomes were compared using Pearson’s χ^2^ test or Fisher’s exact test for categorical variables and the Wilcoxon rank-sum test for continuous variables. All P values were two-sided, and P < 0.050 was considered statistically significant.

## Results

### Patient characteristics

We retrospectively reviewed the data of 86 patients (43 with RAMIE and 43 with MIE). Before matching, the MIE group exhibited a significantly higher clinical T factor (UICC 7th ) and a significantly greater number of cases receiving preoperative therapy. No other baseline characteristics differed significantly between the two groups.

A 1:1 PSM was performed to generate matched pairs and reduce selection bias. This strategy resulted in 26 matched pairs each, and most baseline data were balanced between the two matched cohorts after PSM. Nevertheless, only the clinical N factor (UICC 7th ) was significantly higher in the RAMIE group (Table 1).

**Table 1:** Demographic and clinical characteristics before and after propensity score matching

Characteristics	Before matching			After matching		
	MIE	RAMIE	P value	MIE	RAMIE	P value
No. of patients (%)	43	43		26	26	
Median age: years (range)	68(50–78)	65(47–78)	0.472	67.5(50–76)	66(47–77)	0.832
<65 / 65–75 / >75	16(37.2)/ 25(58.1)/2(4.7)	21(48.8)/19(44.2)/3(7.0)		9(34.6)/16(61.5)/1(3.8)	9(34.6)/15(57.7)/2(7.7)	
Sex			0.750			0.574
Male / Female	37(86.0)/6(14.0)	38(88.4)/5(11.6)		23(88.5)/3(11.5)	24(92.3)/2(7.7)	
BMI	20.7(16–29)	21.7(18–28)	0.830	20.8(15.7–28.8)	23.0(18.1–28.3)	0.376
<18.5 / 18.5 < BMI < 25 / >25	6(14.0)/32(74.4)/5(11.6)	5(11.6)/33(76.7)/5(11.6)		4(15.4)/19(73.1)/3(11.5)	3(11.5)/18(69.2)/5(19.2)	
Smoking (Brinkman Index)			0.791			0.587
None / <400 / >400	9(20.9)/2(4.7)/32(74.4)	8(18.6)/2(4.7)/33(76.7)		4(15.4)/0(0)/ 22(84.6)	5(19.2)/1(3.8)/20(76.9)	
Alcohol			0.301			1.000
None / Opportunity / Habitual	4(9.3)/9(20.9)/30(69.8)	3(7.0)/5(11.6)/35(81.4)		3(11.5)/3(11.5)/20(76.9)	3(11.5)/3(11.5)/20(76.9)	
ASA			0.466			0.381
1 / 2 / 3	13(30.2)/27(62.8)/3(7.0)	17(39.5)/23(53.5)/3(7.0)		11(42.3)/14(53.8)/1(3.8)	8(30.8)/16(61.5)/2(7.7)	
Myocardial infarction	0(0)	1(2.3)	0.323	0(0)	0(0)	-
Heart failure	0(0)	1(2.3)	0.323	0(0)	0(0)	-
Cerebrovascular disease	2(4.7)	3(7.0)	0.650	1(3.8)	2(7.7)	0.574
Liver disease	1(2.3)	0(0)	0.323	0(0)	0(0)	-
Diabetes without chronic complications	4(9.3)	2(4.7)	0.403	2(7.7)	2(7.7)	1.000
Tumor Location			0.617			0.559
Upper / Middle / Lower third	5(11.6)/16(37.2)/22(51.2)	2(4.7)/19(44.2)/22(51.2)		3(11.5)/11(42.3)/12(46.2)	2(7.7)/10(38.5)/14(53.8)	
Histology			0.650			0.574
SCC / Adeno	40(93.0)/3(7.0)	41(95.3)/2(4.7)		25(96.2)/1(3.8)	24(92.3)/2(7.7)	
Clinical T factor			0.002			0.381
1b / 2 / 3	19(44.2)/8(18.6)/16(37.2)	31(72.1)/8(18.6)/4(9.3)		15(57.7)/6(23.1)/5(19.2)	18(69.2)/4(15.4)/4(15.4)	
Clinical N factor			0.085			0.032
0 / 1 / 2	35(81.4)/8(18.6)/0(0)	29(67.4)/12(27.9)/2(4.7)		24(92.3)/2(7.7)/0(0)	18(69.2)/7(26.9)/1(3.8)	
Clinical TNM stage			0.139			0.802
I / II / III	18(41.9)/19(44.2)/6(14.0)	23(53.5)/18(41.9)/2(4.7)		15(57.7)/9(34.6)/2(7.7)	14(53.8)/10(38.5)/2(7.7)	
Preoperative therapy	6(14.0)	2(4.7)	0.010	2(7.7)	2(7.7)	1.000
None / NAC (FP) / NAC (FP) RT	21(48.8)/14(32.6)/8(18.6)	29(67.4)/11(25.6)/3(7.0)		18(69.2)/5(19.2)/3(11.5)	18(69.2)/5(19.2)/3(11.5)	

BMI: Body mass index, ASA: American Society of Anesthesiologists, SCC: Squamous cell carcinoma, Adeno: Adenocarcinoma, NAC: Neoadjuvant chemotherapy, FP: 5-FU + Cisplatin, NACRT: Neoadjuvant chemoradiotherapy

### Short-term postoperative outcomes

The short-term postoperative outcomes of the two groups are presented in Table 2. There was no significant difference in the number of dissected thoracic lymph nodes between the unmatched or matched groups. Before PSM, the operation time (min) was significantly longer in the RAMIE group than in the MIE group (P = 0.019). Moreover, the blood loss volume (mL) was significantly lower (P < 0.001) in the RAMIE group. The number of patients undergoing three-FLND was significantly fewer (P = 0.028) in the RAMIE group. The number of postoperative complications (Clavien–Dindo: CD ≥ 2) was significantly lower in the RAMIE group (P = 0.04), and postoperative hospital stay was significantly shorter in the RAMIE group than in the MIE group (P < 0.001).

**Table 2:** Intraoperative and postoperative outcomes

	Before matching			After matching		
	MIE	RAMIE	P value	MIE	RAMIE	P value
No. of patients (%)	43	43		26	26	
Thoracic operative time (min) mean	229(128–460)	250(162–339)	0.019§	240.5(148–460)	241.5(193–339)	0.110#
Blood loss (ml) mean	65(0–446)	26(0–600)	< 0.001§	62.5(0–446)	31.5(0–355)	0.012#
3-FLND	13(30.2)	4(9.3)	0.028‡	7(26.9)	4(15.4)	0.508¶
Dissected thoracic lymph nodes	25(5–60)	24(8–58)	0.638§	28(5–60)	25(10–57)	0.404#
Postoperative hospital stay	27(18–102)	13(8–40)	< 0.001§	28(18–102)	14(8–35)	< 0.001#
Pathological T factor			0.002‡			0.200
Tis, 1a / 1b / 2 / 3	1(2.3)/23(53.5)/3(7.0)/16(37.2)	5(11.6)/31(72.1)/5(11.6)/2(4.7)		1(3.8)/17(65.4)/1(3.8)/7(26.9)	3(11.5)/18(69.2)/3(11.5)/2(7.7)	
Pathological N factor			0.375‡			0.023
0 / 1 / 2	27(62.8)/15(34.9)/1(2.3)	26(60.5)/13(30.2)/4(9.3)		18(69.2)/8(30.8)/0(0)	19(73.1)/5(19.2)/2(7.7)	
Pathological TNM stage			0.465‡			0.306
0 / I / II / III	10(23.3)/9(20.9)/14(32.6)/10(23.3)	15(34.9)/11(25.6)/11(25.6)/6(14.0)		8(30.8)/6(23.1)/8(30.8)/4(15.4)	11(42.3)/7(26.9)/5(19.2)/3(11.5)	
Postoperative complication (CD ≧ II)	20(46.5)	10(23.3)	0.04	13(50.0)	5(19.2)	0.021¶
Pneumonia	5(11.6)	3(7.0)	0.713‡	2(7.7)	1(3.8)	1.000¶
Anastomotic leakage			0.110‡			0.125¶
Type I / Type II	0(0)/6(14.0)	0(0)/1(2.3)		0(0)/4(15.4)	0(0)/0(0)	
RLN paralysis			0.112			0.070¶
I	12(27.9)	6(14.0)		10(38.5)	4(15.4)	
Chylothorax			1.000‡			-
Type I	0(0)	1(2.3)		0(0)	0(0)	
Recurrence	11(25.6)	10(23.3)	0.802	6(23.1)	6(23.1)	1.000¶

MIE: minimally invasive esophagectomy, RAMIE: robot-assisted minimally invasive esophagectomy, FLND: Field lymphatic node dissection, CD: Clavien–Dindo classification, RLN: Recurrent laryngeal nerve

‡Pearson’s χ2 test or Fisher’s exact test, except §Wilcoxon rank-sum test

¶McNemar’s test or McNemar’s exact test, except #Wilcoxon signed-rank test

After PSM, the RAMIE group had a significantly lower amount of blood loss (mL) (P = 0.012), fewer postoperative complications (CD ≥ 2) (P = 0.021), and shorter postoperative hospital stay (P < 0.001) than the MIE group. There was no significant difference in the number of three-FLND, lymph node dissections, and incidence of major complications, such as anastomotic leakage (P = 0.125), RLN paralysis (P = 0.070), and pneumonia (P = 1.000), between the unmatched and matched groups.

### Patient survival and disease recurrence

#### Long-term outcomes

**Fig. 2:**
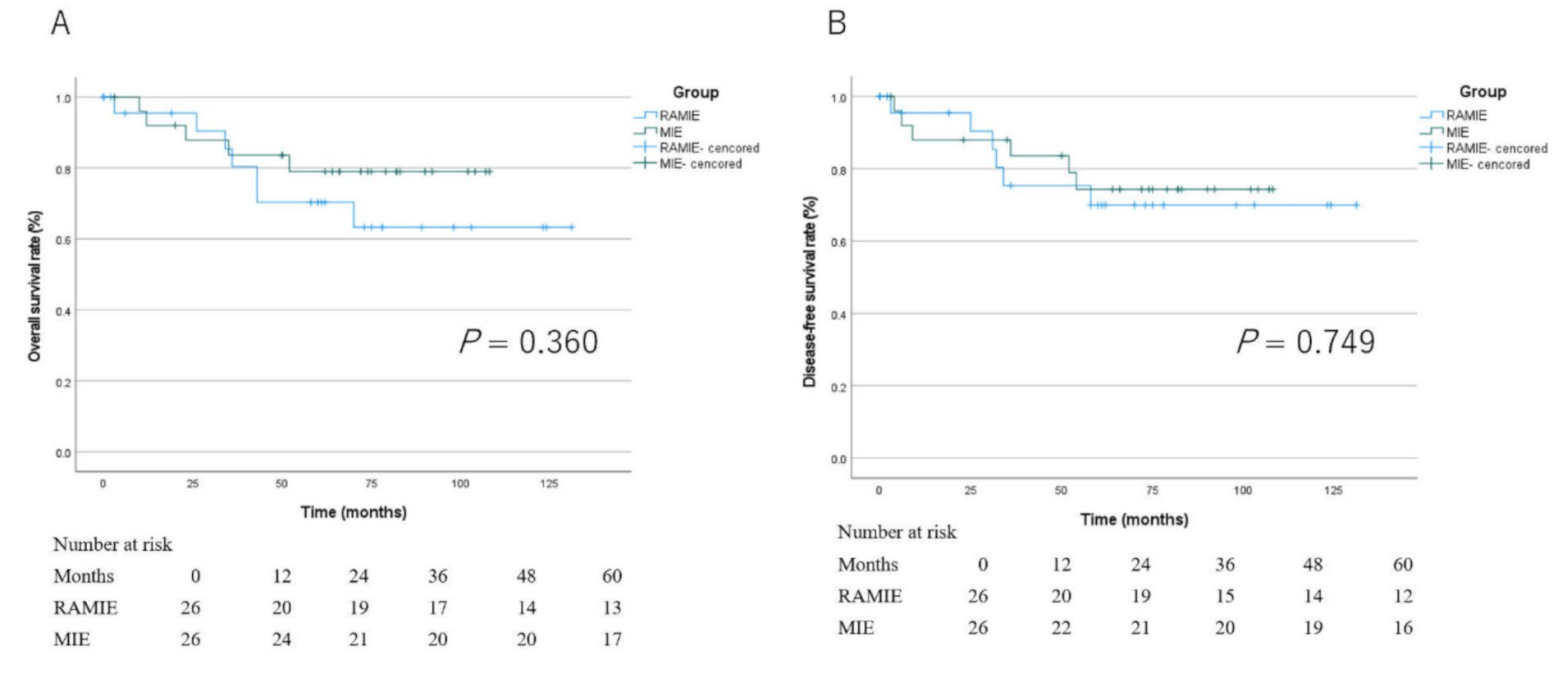
Overall and disease-free survival analysis in the two matched cohorts. (**A**) OS, (**B**) DFS. OS: overall survival, DFS: disease-free survival

After PSM, 52 patients were included in survival analysis. The median observation period was 59 months (range, 0–131 months) in the RAMIE group and 69 months (range, 3–108 months) in the MIE group. The 5-year OS rates in the RAMIE group and MIE group were 73.1% (95% CI: 76–117) and 80.8% (95% CI: 77–104), respectively (P = 0.360). The Kaplan–Meier curves for OS in both groups are shown in Fig. 2. The DFS for the RAMIE group was 58 months (range, 0–131 months), and the median DFS was 69 months (range, 3–108 months) for the MIE group. The 5-year DFS rates were 76.9% (95% CI: 80–121) in the RAMIE group and 76.9% (95% CI: 72–102) in the MIE group (P = 0.749). The Kaplan–Meier curve for DFS is shown in Fig. 2.

There was no significant difference in the 5-year OS and DFS rates between the RAMIE and MIE groups (Fig. 2). The incidence of tumor recurrence, including lymph nodes (only inside the resection area, only outside the resection area, and both areas) and distant areas (lung, liver, cerebral, adrenal, dissemination), was compared between patients in the matched cohorts. As shown in Table 3, total recurrences were observed in six (23.1%) and six (23.1%) patients in the two matched cohorts after PSM (P = 1.000).

**Table 3:** Location of recurrence and cause of death

	Before matching			After matching		
	MIE	RAMIE	P value‡	MIE	RAMIE	P value¶
No. of patients (%)	43	43		26	26	
Overall recurrence disease	11(25.6)	10(23.3)	0.802	6(23.1)	6(23.1)	1.000
Anastomoses/gastric conduit	0(0)	0(0)		0(0)	0(0)	
lymph nodes	7(16.3)	6(14.0)	0.763	4(15.4)	3(11.5)	1.000
Only inside the resection area	6(14.0)	2(4.7)	0.133	3(11.5)	2(7.7)	1.000
Only outside the resection area	1(2.3)	3(7.0)	0.308	1(3.8)	1(3.8)	1.000
Both	0(0)	1(2.3)	0.500	0(0)	0(0)	
Distant	6(14.0)	4(9.3)	0.501	3(11.5)	3(11.5)	1.000
Lung	4(9.3)	1(2.3)	0.180	2(7.7)	1(3.8)	1.000
Liver	1(2.3)	3(7.0)	0.308	0(0)	2(7.7)	0.500
Cerebral	1(2.3)	0(0)	0.500	1(3.8)	0(0)	1.000
Adrenal	0(0)	1(2.3)	0.500	0(0)	0(0)	
Dissemination	0(0)	1(2.3)	0.500	0(0)	1(3.8)	1.000
No. of patients						
Death	10(23.3)	11(25.6)	0.802	5(19.2)	6(23.1)	0.727
Cancer	8(18.6)	9(20.9)	0.417	3(11.5)	5(19.2)	0.508
Other diseases	2(4.7)	2(4.7)	1.000	2(7.7)	1(3.8)	1.000

MIE: minimally invasive esophagectomy, RAMIE: robot-assisted minimally invasive esophagectomy

‡Pearson’s χ2 test or Fisher’s exact test, ¶McNemar’s test or McNemar’s exact test

The median recurrence periods were 41.5 (range, 3–58) months in the RAMIE group and 22.5 (range, 4–54) months in the MIE group. In the RAMIE group, three (11.5%) patients had lymph node recurrence (only inside the resection area: 2, only outside the resection area: 1), and 3 (11.5%) patients had distant recurrence (lung: 1, liver: 2). In the MIE group, 4 (15.4%) patients had lymph node recurrence (only inside the resection area: 3, only outside the resection area: 1), and 3 (11.5%) patients had distant recurrence (lung: 2, cerebral: 1). There was no significant difference in the recurrence rates between the two matched groups.

## Discussion

The present study demonstrated the clinical feasibility and potential clinical advantages of RAMIE compared with that of MIE. To our knowledge, this is the first report describing the long-term outcomes of RAMIE in Japan. Although some studies have reported that the operative time did not differ between RAMIE and MIE [9, 18], several demonstrated that RAMIE requires a significantly longer time [10, 11, 24]. This may be owing to the time required for the roll-in and docking of the da Vinci used in RAMIE. In our study, this difference was not statistically significant after PSM because multiple RAMIE experiments seem to have shortened the Vinci roll-in and docking. The introduction of da Vinci Xi further facilitates this process.

There is a tendency toward less intraoperative blood loss in RAMIE than in MIE [9–11, 24, 25]. In our study, intraoperative blood loss was significantly lower in the RAMIE group. Intricate operations are generally possible using forceps with a joint with a high degree of freedom under 3-dimensional imaging that clearly shows the depth. These elements could aid RAMIE and contribute to a reduction in blood loss.

Therefore, it may be possible to perform delicate procedures for lymph node dissection using RAMIE. Lymph node dissection around the RLN is crucial in esophagectomy, with a key focus on preserving RLN integrity. Several lymph nodes around the RLN are frequently dissected during RAMIE, and the frequency of postoperative RLN paralysis is equivalent to that of MIE [10, 11, 16, 24, 26]. Some studies have reported no significant differences in the number of dissected lymph nodes, as observed in the present study. Nevertheless, several studies have reported a significantly lower frequency of postoperative RLN paralysis after RAMIE. Suda et al. reported that the use of a surgical robot that promotes accurate RLN identification and dissection can reduce the risk of RLN injury, resulting in preserved laryngopharyngeal function [9].

Furthermore, Park et al. [18] reported that highly efficient lymph node dissection is possible using RAMIE. No significant difference in the number of dissected lymph nodes between the RAMIE and MIE groups was noted; however, the frequency of postoperative RLN paralysis was lower in the RAMIE group. These results indicated the usefulness of RAMIE for lymph node dissection around the RLN.

Several studies have indicated comparable postoperative complications and postoperative hospital stay durations for RAMIE and MIE [10, 11, 18, 24–26]. However, RAMIE resulted in significantly fewer postoperative complications (CD ≥ 2) in our study, and the frequency of RLN paralysis tended to be lower in the RAMIE group. The advantage of RAMIE in intricate procedures has been proposed, making it potentially superior to MIE in mitigating RLN paralysis. In addition, postoperative hospital stay was significantly shorter in the RAMIE group than in the MIE group. The reduction in recurrent nerve palsy and anastomotic leakage has led to a reduction in the postoperative hospital stay. In contrast, several patients in the MIE group required long-term rehabilitation because of RLN paralysis, and this may have resulted in a significant difference in the duration of hospital stay between the two groups. Moreover, the incidence of anastomotic leakage significantly differed between the two groups. The variations in the number of hospital days may be attributed to the longer duration required for treating suture failure in comparison to robotic surgery.

Recently, some studies have compared the long-term results of RAMIE to those of MIE and open esophagectomy [16–18], reporting no significant differences in OS and DFS. Similarly, no significant differences were noted in the 5-year OS and DFS rates between the RAMIE and MIE groups. Nevertheless, the MIE group had a higher percentage of clinical N0 factor compared to the RAMIE group following PSM. These factors might have significantly impacted the survival data. However, there was no difference in the percentages of pathological N0 factor for the MIE and RAMIE groups.

As aforementioned, both our study and prior reports suggest the superiority of RAMIE to MIE with regard to blood loss and RLN-related lymph node dissection. In addition, no study has reported that the long-term outcomes of RAMIE are less favorable than those of thoracotomy and MIE. Furthermore, learning the RAMIE technique, given its short learning curve, is feasible even for less-experienced MIE operators [7, 21], highlighting another valuable characteristic of RAMIE. With these insights, the RAMIE could establish itself as the standard for esophageal cancer treatment. Enhancements in surgical robot technology, including cost, size, and weight reduction, and the development of new surgical robots by various companies will be important for further advancements.

This study has several limitations. First, this was a retrospective, single-center investigation with a small number of patients. Second, the decision for preoperative treatment was based on the discretion of the oncologists and surgeons and patient preference. Third, the majority of the study population had squamous cell carcinoma. The survival effect of preoperative treatment may differ in patients with adenocarcinoma.

An external validation study involving a sufficient number of patients is required to confirm our findings.

## Conclusions

On comparing the short- and long-term outcomes of RAMIE and MIE using PSM, we demonstrated that blood loss and postoperative complications were significantly lower, and the duration of hospital stay was significantly shorter with RAMIE than with MIE. No significant differences were observed in long-term outcomes (5-year OS and DFS) between the RAMIE and MIE groups. Our results suggested that RAMIE can be used as the standard surgical procedure for the treatment of esophageal cancer in the future. Robotic-assisted surgery will continue to evolve, and if its safety and usefulness are established through further data collection and analyses in randomized controlled trials, robotic surgery will soon become a standard procedure.

## Data Availability

The datasets used and/or analysed during the current study are available from the corresponding author on reasonable request.

## Abbreviations

DFS: Disease free survival
HALS: Hand assisted laparoscopic surgery
MIE: Minimally invasive esophagectomy
OS: Overall survival
PSM: Propensity score matching
RAMIE: Robot-assisted minimally invasive esophagectomy
RLN: Recurrent laryngeal nerves
SMD: Standardized mean difference
FLND: Field lymph node dissection
