# Correction: Long-term outcomes of robot-assisted versus minimally invasive esophagectomy in patients with thoracic esophageal cancer: a propensity score-matched study

**DOI:** 10.1186/s12957-025-03689-2

**Published:** 2025-02-04

**Authors:** Toru Sakurai, Akihiro Hoshino, Kenta Miyoshi, Erika Yamada, Masaya Enomoto, Junichi Mazaki, Hiroshi Kuwabara, Kenichi Iwasaki, Yoshihiro Ota, Shingo Tachibana, Yutaka Hayashi, Tetsuo Ishizaki, Yuichi Nagakawa

**Affiliations:** 1 Department of Gastrointestinal and Pediatric Surgery, Tokyo Medical University, 6-1-1 Nishi-Shinjuku, Shinjuku-Ku, Tokyo, 160-0023 Japan; 2 Department of Digestive Surgery, Kohsei Chuo General Hospital, 1-11-7 Mita, Meguro-Ku, Tokyo, 153-8581 Japan; 3 Department of Surgery, Toda Chuo General Hospital, 1-19-3 Hon-Chou, Toda, Saitama 335-0023 Japan

**Correction** **:** **World J Surg Onc 22, 80 (2024)**

**https://doi.org/10.1186/s12957-024-03358-w**

Following publication of the original article [1], the author reported that in Figure 2, they incorrectly calculated the number alive and recurrence free at each time point. There is no changes in the image but in the data below the image.

Old figure


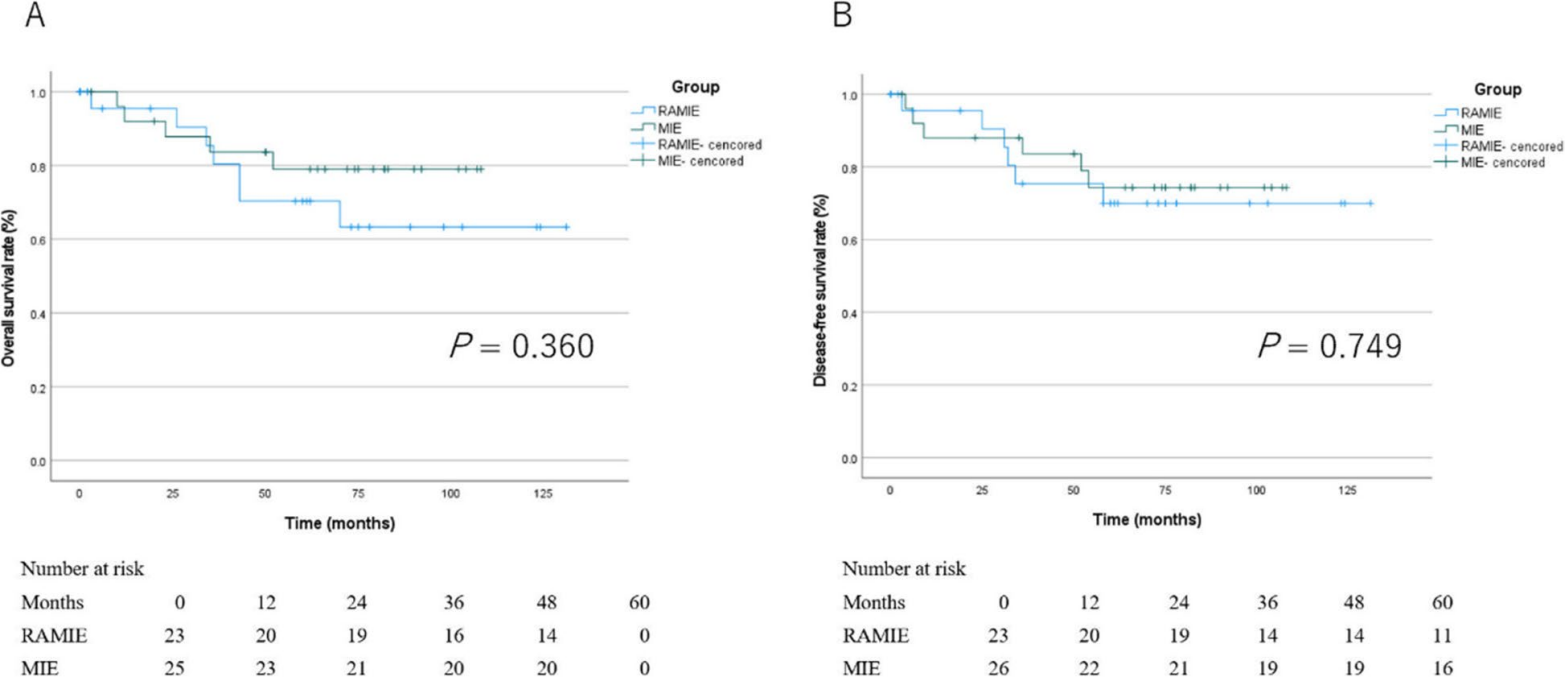


New Figure


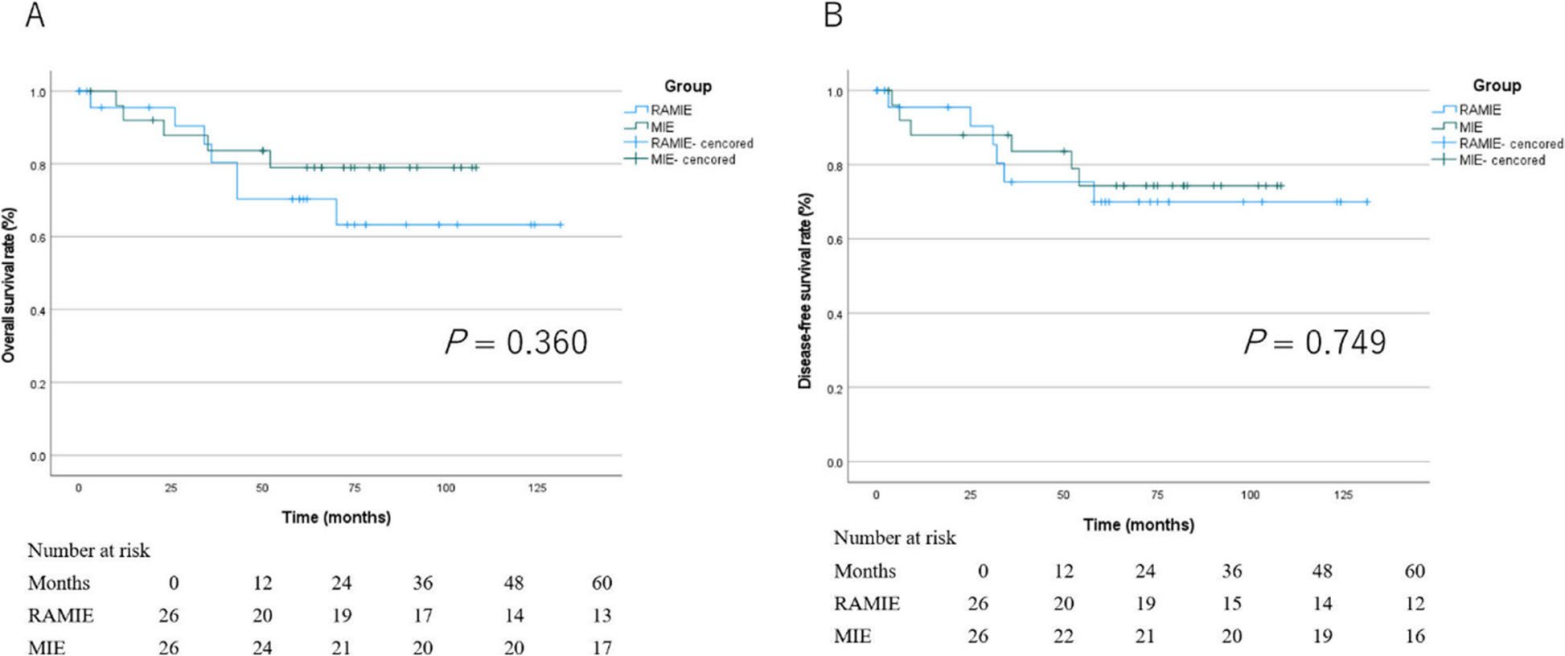


The original article has been updated.
